# Junction region of EWS-FLI1 fusion protein has a dominant negative effect in Ewing’s Sarcoma *in vitro*

**DOI:** 10.1186/1471-2407-12-513

**Published:** 2012-11-12

**Authors:** Babu Jully, Ramshankar Vijayalakshmi, Gopisetty Gopal, Kesavan Sabitha, Thangarajan Rajkumar

**Affiliations:** 1 Department of Molecular Oncology, Cancer Institute (WIA), 38, Sardar Patel Road, Chennai, 600036, India

## Abstract

**Background:**

Ewing’s sarcoma is a malignancy characterized by a specific 11:22 chromosomal translocation which generates a novel EWS-FLI1 fusion protein functioning as an aberrant transcription factor. In the present study, we have further characterized the junction region of the EWS-FLI1 fusion protein.

**Methods:**

In-silico model of EWS-FLI1 fusion protein was analysed for ligand binding sites, and a putative region (amino acid (aa) 251–343 of the type 1 fusion protein) in the vicinity of the fusion junction was cloned and expressed using bacterial expression. The recombinant protein was characterized by Circular Dichroism (CD). We then expressed aa 251–280 ectopically in Ewing’s sarcoma cell-line and its effect on cell proliferation, tumorigenicity and expression of EWS-FLI1 target genes were analysed.

**Results:**

Our modelling analysis indicated that Junction region (aa 251–343) encompasses potential ligand biding sites in the EWS-FLI1 protein and when expressed in bacteria was present as soluble form. Ectopically expressing this region in Ewing’s sarcoma cells inhibited tumorigenicity, and EWS-FLI1 target genes indicating a dominant negative biological effect.

**Conclusions:**

Junction region can be exploited further as target for drug development in future to specifically target EWS-FLI1 in Ewing’s Sarcoma.

## Background

Ewing’s sarcoma is a highly malignant bone and soft tissue tumor occurring in children and young adults. More than 85% of the Ewing’s sarcoma family of tumours (ESFT) patients present with a balanced t(11:22) (q24;q12) chromosomal translocation [1,2]. This reciprocal translocation generates a novel in frame fusion gene with a unique junctional region between sequences which encode the N-terminus of the RNA binding protein EWS from chromosome 22 and the C-terminus of FLI1 transcription factor on chromosome 11 [3,4]. Several evidences have shown EWS-FLI1 as a well described oncogene and with depletion of this gene product resulting in inhibition of ESFT growth. EWS-FLI1 fusion protein therefore is a validated tumor target functioning as an aberrant transcription factor [4,5]. Transforming activity of EWS-FLI1 requires both the EWS portion of the fusion protein which contributes to transactivation and the ETS domain (FLI1 portion) which mediates sequence-specific DNA binding [6-8].

Structure of EWS-FLI1 is not available in the PDB and it is an intrinsically disordered Protein (IDP). These kind of proteins are insoluble, unstructured and do not have specific Ramachandran angles in the protein backbone and show polymorphism in bound state [9,10].

In this study, we looked at EWS-FLI1 protein structure using modelling tool and bioinformatics tools to analyze potential structure in-silico. Our analysis indicated potential ligand binding sites which encompass the junction region of the EWS-FLI1 protein and that the region was likely to have a structure indicated by alpha helical and beta pleated structures. The junction region (aa 251–343) containing type 1 fusion residues was expressed and purified and subjected to circular dichroism (CD) analysis. Finally our analysis of the biological effects of ectopically expressing junction region on expression of EWS-FLI1 target genes, and proliferation of Ewing’s sarcoma cells in-vitro indicates a dominant negative function for the junction region.

## Methods

### RNA Extraction and cDNA preparation

The study was approved by the Cancer Institute Ethics Committee. RNA was isolated from biopsy sample from a patient diagnosed with Ewing’s Sarcoma after an informed consent. RNA was qualitatively and quantitatively assessed on 1.25% agarose gel and by spectrophotometer. cDNA was synthesized using Superscript II (Invitrogen) as per manufacturer’s instructions and β actin amplification was done to check its quality. Full length EWS-FLI1 Type1 fusion gene was amplified. The PCR product was purified and sequenced using ABI 310 Genetic analyzer. This sequence was submitted in GenBank [GenBank: ACA62796].

### In-silico analysis of EWS-FLI1 protein using prime and SiteMap

Prime (Version 3.0, Schrodinger, LLC, New York) was used to build EWS-FLI1 structure. The OPLS2000 all-atom force field was used for energy scoring of proteins. Surface Generalized Born (SGB) continuum solvation model, was used for treating solvation effects; and side chain rotomer and backbone dihedral libraries derived from PDB non-redundant structures were used for building backbone and side chains. The modelled structure was imported and corrections were carried out by Protein Preparation wizard of Schrodinger, where hydrogens were added automatically and refinement of the structure was done. EWS-FLI1 protein was assessed for putative ligand binding sites using SiteMap. The programme highlights regions within the protein suitable for occupancy by hydrophobic groups or by ligand hydrogen-bond donors, acceptors, or metal-binding functionality. SiteScore, the scoring function was used to assess a site's propensity for ligand binding, and rank possible binding sites.

### Cloning and expression of EWS-FLI1

The sequenced EWS-FLI1 Type 1 fusion gene was cloned in pET 102/D-TOPO vector (Invitrogen) using the primer set PET 102/D-TOPO Forward. 5’CACCATGGCGTCCACGGATT3’ and Reverse. 5’GTAGTAGCTGCCTAAGTGTGA 3’. Full length EWS-FLI1 type 1 c-DNA was cloned into pCR2.1 using TA cloning method. The insert was then subcloned into pGEX-KG a GST fusion vector to be expressed as GST fusion protein. Ligation mixtures of EWS-FLI1 in pET 102/D TOPO and pGEX-KG were used to transform E.coli TOP 10, chemically competent cells and selected in ampicillin (100 μg/ml) containing medium. The positive clones were determined by colony PCR and sequencing. Full length EWS-FLI1 was cloned into pGEX-KG and pET 102/D-TOPO vector were expressed in BL21-CodonPlus(DE3)-RP competent cells (Stratagene, La Jolla, CA) containing extra copies of ArgU and ProL genes to overcome the codon bias [11]. Protein production was initiated by adding 0.4 mM isopropyl β-D-1- thio galactopyranoside (IPTG), and bacteria were cultured for an additional 3–4 h at 20°C.

### Immunoblotting

Lysates from Bacteria cells expressing Thioredoxin (Trx)-EWS-FLI1-His tagged protein, whole cell lysates from MCF-7, EWS502 cells stably expressing pCDNA/FLAG and pCDNA/FLAG/Junction (aa 251–280) constructs were used for immunoblot analysis. The antibodies used were anti-FLI1 (C-19) antibody (Santa Cruz Biotechnology, Inc., Santa Cruz, CA, USA) , E Cadherin (abcam, Cambridge, UK), Vimentin (abcam, Cambridge, UK), Beta Actin (Sigma, Saint Louis, MI, USA). Bacteria cells expressing Thioredoxin (Trx)-EWS-FLI1-His tagged protein, suspended in native lysis buffer (20 mM Tris pH 8, 500 mM NaCl, 0.1%NP 40, Lysozyme 1mg/ml), subjected to lysis by sonication and centrifuged at 12000 rpm. The supernatant (soluble) and pellet (insoluble) fraction were analysed in SDS PAGE (10%). Lysates from MCF-7, and EWS502 cells stably expressing pCDNA/FLAG or pCDNA/FLAG/Junction (aa 251–280) constructs were prepared in RIPA buffer (1% Nonidet P-40, 1% sodium deoxycholate, 0.1% SDS, 0.15M Sodium chloride, 0.05M Tris.Cl, pH8.0). 50 micro grams of the whole cell lysate was used for the analysis. Gels were transferred onto poly vinylidene difluoride (PVDF) membrane (Millipore, Billerica, MA, USA). This filter was blocked with 5% non-fat milk in TBS-T (20 mM Tris–HCl, pH 7.5, 150 mM NaCl, 0.1% Tween 20) for 1 hr at room temperature and then probed with respective primary and secondary antibody. The blot was incubated and visualized with enhanced chemiluminescence (ECL) solution (GE Healthcare Life Sciences, Buckinghamshire, UK) according to the manufacturer’s instructions.

### Purification of recombinant EWS-FLI1

Trx-Full length EWS-FLI1-6xHis tagged Protein was purified by Ni-NTA immobilized metal-ion affinity chromatography (IMAC) under denaturing conditions. Lysis/Equilibration buffer/wash buffer compositions: 50 mM Na_3_PO_4_ (pH 7.8) containing 500 mM NaCl, 20 mM Tris HCl, 6 M Urea and Elution buffer: wash buffer containing 200mM imidazole. Initially, precharged metal chelating columns were washed twice with 10 mL of distilled water, and equilibrated in 10 mL of equilibration buffer. Cell pellet corresponding to 100 mL of bacterial culture was re-suspended in 10 mL of lysis buffer. Debris was removed by centrifugation at 4000*g* for 10 min at 4°C. The supernatant was applied to Ni-NTA affinity column (Invitrogen, Carlsbad, CA, USA ) and incubated at room temperature for 30 minutes with constant rocking [12]. Column was washed three times and protein was then eluted from the column using 200mM imidazole. Aliquots (1 mL) were collected and a portion of which was then applied to 10% polyacrylamide gel for Coomassie staining and immunoblotting.

### Tryptic mapping and mass spectrometry

Mass spectrometry of the purified EWS-FLI1 was performed at the Mass Spectroscopy Facility at The Centre for Genomics Research, New Delhi, India.

### Fold index plot

Amino acid sequence of EWS-FLI1 (submitted in GenBank) was assessed to identify the ordered and disordered region within the protein using Fold Index predictor available as a graphic web server [13,14].

### Purification of soluble junction construct

Junction (aa 251 – 343) region was cloned in pET 102/D-TOPO vector (Invitrogen Carlsbad, CA, USA) using primer set Junction (aa 251 – 343) Forward. 5’CACCCCAAGTCAATATAGCAACAGAGC 3’, and Junction (aa 251 – 343) Reverse. 5’GGCGTTGGCGCTGTCGGAG 3’ and expressed in BL21-CodonPlus (DE3)-RP competent cells. Expression of Trx-Junction (aa 251–343) -6xHis tagged protein was confirmed by immunoblotting using anti-thio antibody and horseradish peroxidase-conjugated anti-mouse antibodies as primary and secondary antibody, respectively. Protein was purified by Ni-NTA immobilized metal-ion affinity chromatography (IMAC) under native condition. Buffer compositions were: Lysis/Binding/Equilibration buffer 50 mM sodium phosphate buffer, 300 mM NaCl, 5 mM imidazole, 0.1%NP-40 pH 8.0. Wash (W1) 50 mM sodium phosphate buffer, 300 mM NaCl, 20 mM imidazole, pH 8.0. Wash (W2) 50 mM sodium phosphate buffer, 300 mM NaCl, 50 mM imidazole, pH 8.0. Elution buffer 50 mM sodium phosphate buffer, 300 mM NaCl, 200 mM imidazole, pH 8.0.

### Dialysis and circular dichroism

Affinity purified Junction protein (aa 251–343) in buffer containing 50 mM sodium phosphate buffer, 300 mM NaCl, 200 mM imidazole, pH 8.0 was concentrated using Centricon concentrator (EMD Millipore, Billerica, MA, USA) with a 10 kDa cut off and dialyzed against 10 mM sodium phosphate buffer, pH 8.0. The concentrated protein (2mg/ml) was subjected to circular dichroism.

### Expression of short junction region (aa 251 to aa 280) in Ewing Sarcoma cells

The short junction construct, a 30 amino acid containing peptide (aa 251 to aa 280) which overlaps EWS-FLI1 junction region was cloned into pcDNA3.1/FLAG vector at EcoRV and XhoI restriction sites using the primer set (aa 251 to aa 280) Forward. 5’GATATCCCAAGTCAAATAACCCAACAGAG 3’ and (aa 251 to aa 280) Reverse 5’CTCGAGCTACATGTTATTGCCCCA 3’. EWS502 Ewing’s sarcoma cell line was grown in RPMI-1640 supplemented with 10 % fetal calf serum. 5 μg of pCDNA3.1/ FLAG-junction and pCDNA3.1/FLAG plasmid alone were transfected separately into EWS502 cell line (harbouring EWS-FLI1 type1 fusion gene) using Fugene (Roche, Molecular Biochemicals, Indianapolis, IN, USA) transfection kit as per the manufacturer’s instructions. The transfected cell line was selected in 100μg/ml hygromycin and maintained in presence of hygromycin. Single clone of transfected cells was isolated by serial dilution. Expression of Flag tagged junction construct in transfected EWS502 cell line was confirmed by western blot.

### Colony forming assay

EWS502 pCDNA/FLAG alone and EWS502 pCDNA/FLAG/junction (aa 251–280) cells were plated in agar in duplicates at density of 5000 cells per well in a 6 well culture plate. Bottom layer was prepared with 0.5% agar and top layer with 0.2% agar. Culture plates were incubated at 37°C and 5% CO2 in a humidified atmosphere. Colonies were enumerated after 3 weeks of growth. All the experiments were repeated at least twice.

### Real-Time quantitative PCR

Real time quantitation of EWS-FLI1 modulated targets NROB1, NKX2.2, GLI1, Cyclin D1, c-MYC, EZH2, TGFβIIR, KFL2 and EMT markers E-Cadherin, Vimentin, Slug, N-Cadherin and Fibronectin was done using SYBR green master mix (Fermentas GMBH, Germany) as per manufacturer’s instructions. Three individual clones of the junction construct (aa 251–280) transfected EWS502 were analyzed for Real time quantitation. Each sample was assessed in triplicate to ensure reproducibility of the quantitative measurements. GAPDH expression was evaluated for each sample as a control for total RNA. The experiments were repeated at least twice.

## Results

### Junction region (aa 251 to aa 343) encompasses potential ligand binding sites in the EWS-FLI1 protein

**Figure 1:**
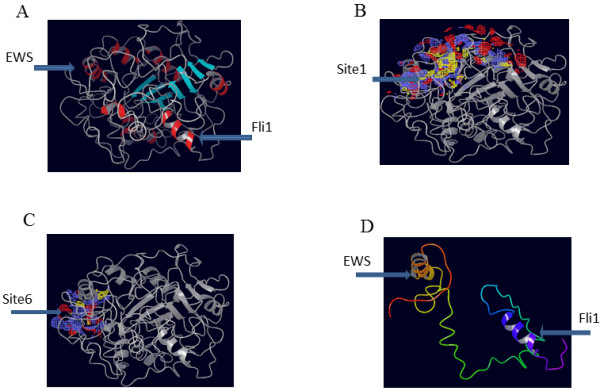
**In-silico structure of EWS-FLI1 protein and putative small molecule binding regions.A**, In-silico structure of EWS-FLI1 protein. Figure 1**B** and **C** model structures denoting the small molecule binding regions. Figure 1**D** the structure of the region form a.a.251- a.a.343 in the vicinity of the junction region of EWS-FLI1 protein.

Since the crystal structure of EWS-FLI1 protein was not available we chose to study the putative structure of the protein modelled using an proprietary algorithm insilico. The protein sequence of EWS-FLI1 accession number ACA62796 was used for modelling. The modelled structure of EWS-FLI1 indicated the presence of alpha helices (30–37, 88–94, 116–120, 124–127, 254–262, 264–268, 326–334, 401–412, 458–465), beta sheets (12–15, 54–57, 105–109, 147,148, 352,353, 377–380, 387–389,425,426) and loops (Figure 1A). We examined the protein structure for potential ligand association sites. Sites which received a score greater than 1.0 indicate that they are likely to bind with ligands. Around 5 sites were identified, of which four sites had a site score greater than 1 and encompassed the junction region amino acids (Table 1). Sites 1 and 6 are depicted in Figure 1B and C. Since junction region amino acids were predicted to be part of the potential ligand binding sites we were interested in further understanding the structure in the vicinity of the junction region. Analysis of the structure of junction region extending between aa 251 and aa 343 comprising of both EWS region and FLI1 potion indicated the presence of alpha helical regions (Figure 1D).

**Table 1:** Putative small molecule binding sites in EWS-FLI1 protein

**SITE**	**Residue Number**	**Score**
4	21,156–167,170,173,176-196,203-207,218- 223 **247,248, 317–327**, 389, 391, 394,447-450,454,498	1.151
3	109-112,117,120,121,124,125,128,129,146-148,150-153,298 305, 307, 308,310,315,317,324,325,327,328,331,332,335,345,347,380,382,383,385-389, 481-490	1.077
2	12-16,54-64,66-69,81-86,89-98,100-103,105,107,109,112,119 – 124, 127 , 128,132-146,272,276,290,296,311	1.047
6	25,65,71-75,77,209,213-217,**236-247,250,254-264,278-286**	1.043
5	1,14-16,29,31,32,35,36,41-44,49,50,54-59,66,67,90,92-97,**261-264,267-272**	1.032
9	150,151,158-160,315,317-329,350,377-380,387-391,456,461,489-492	1.026
1	22,25-34,36-40,163,188,190-211,214,216-218,**246-254,257-259, 261,262,264,279-282,318**,352,365-369,377,378,391-401,436,439-450,454	1.007
8	179-181,184,206-208,216,219-223,229-237,**240,244,246,285,288,289**	0.944
7	11,104,106,144-147,307-314,374,383-388,410-413,476,479-482,484	0.948
10	61-63,80-86,89,118,119,223,234,235,288-296	0.777

The table lists the binding sites along with the amino acid residues which form the sites. Residues numbered in bold denote the region in the vicinity of the junction of EWS-FLI1 protein.

**Figure 2:**
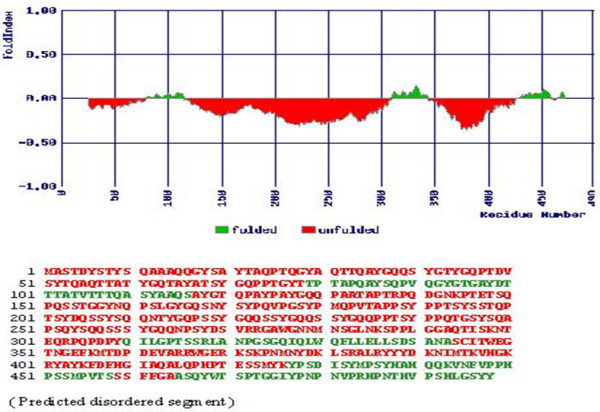
**Bioinformatics based Fold Index values for EWS-FLI1 type 1 schematic translocation.** Fold index plot shows the differentiation between the ordered (upper case: green colored) and disordered (lower case: red colored) based on the amino acid composition. The plot shows extensive unfolded regions (below the 0 line threshold fold index) for the EWS-FLI1 fusion protein based on the average residue hydrophobicity and net charge of the sequence.

Further when analyzed using Fold index tool, the region in the FLI1 portion of the junction indicated a stable region which however did not extend into the EWS portion (Figure 2). Since our modeling studies indicated a functional role as a putative ligand binding region and presence of structured regions in the junction region (aa 251–343) we proceeded to express and purify the junction region.

### Full Length protein is insoluble where as the junction region (aa 251 to aa 343) is soluble

**Figure 3:**
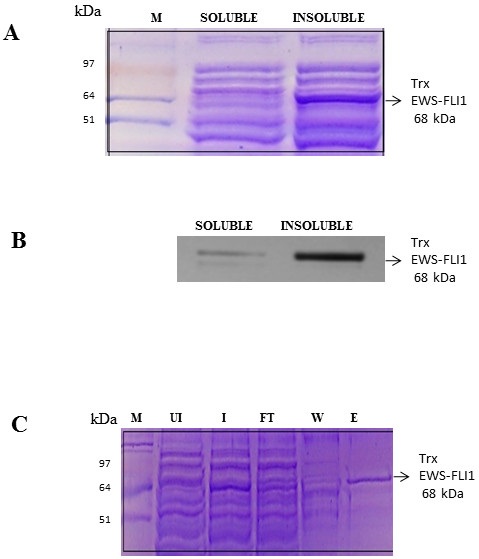
**Bacterial expression and purification of recombinant full length EWS-FLI1.A**: Coomassie stained SDS PAGE gel picture showing,expression of Full Length Trx -EWS-FLI1-His tag Protein in insoluble fraction.Lane 1 represents protein ladder, Lane2 represents soluble fraction , Lane 3 represents insoluble fraction. **B**: Confirmation of EWS-FLI1 protein expression by immunoblotting with anti FLI1 antibody. Lane 1: soluble fraction, Lane 2:insoluble fraction. **C**: Coomassie stained SDS PAGE gel picture of Ni-NTA Purification of full length EWS-FLI1 protein in denaturing condition.Lane1: Protein ladder Lane 2: Uninduced cell lysate, Lane 3: Induced cell lysate , Lane 4: Flow Through, Lane 5: Wash and Lane 6: Elute.

**Figure 4:**
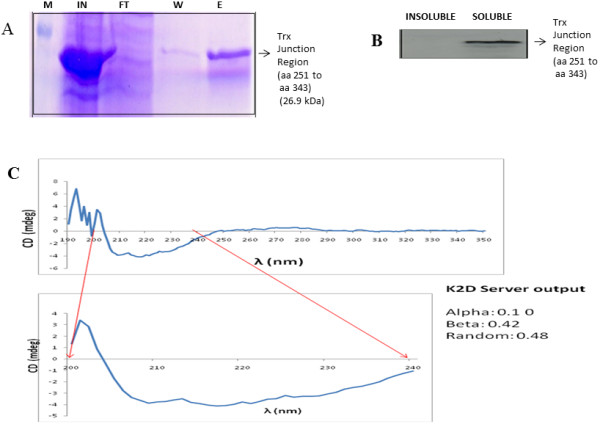
**Bacterial expression and purification of Junction region (aa 251- aa 343).A**: Coomassie stained SDS PAGE gel picture of Ni-NTA Purification of Junction- EWS-FLI1 protein under native condition. Lane1: Protein ladder, Lane 2: Induced cell lysate soluble fraction, Lane 3: Flow Through, Lane 4: Wash and Lane 5: Elute. Figure 4**B**: Confirmation of junction region (aa 251–343) protein expression by immunoblotting with anti Thio antibody. Lane 1: insoluble fraction, Lane 2: soluble fraction. Figure 4**C**: CD of junction region (aa 251-aa 343) of EWS-FLI1 soluble construct.

To start with we expressed the full length recombinant EWS-FLI1 protein with a thioredoxin (Trx) tag in the amino terminus and 6xHis-Tag in the carboxy terminus was expressed in BL21-CodonPlus (DE3)-RP cells. A band consistent with EWS-FLI1 (68 kDa) was observed in the insoluble fraction (Figure 3A) which was confirmed by western blot using Anti FLI1 antibody (Figure 3B). The insoluble fraction of EWS-FLI1 was purified using Ni-NTA purification system in the denaturing condition (Figure 3C). Similarly, GST-EWS-FLI1 could not be taken further for native affinity purification because of its insolubility. The junction region aa 251–343 identified from our modeling studies to be relatively structured was cloned and expressed in a similar manner to the full length protein. The junction region was expressed in soluble fraction of the lysate. Expression of soluble junction region (aa 251–343) was confirmed by Western blot (Figure 4A). Junction region was purified in native condition using Ni-NTA purification system (Figure 4B), concentrated and circular dichroism analysis was performed. The CD readings were taken for the wavelength range of 190-350nm showed highly coiled form (random)- 48%, Beta sheets- 42% and alpha helix 10% (Figure 4C) indicating the presence of structured regions in the protein.

### Ectopic expression of junction construct (aa 251 to aa 280) represses tumorigenicity and alters the expression of EWS-FLI1 target genes

**Figure 5:**
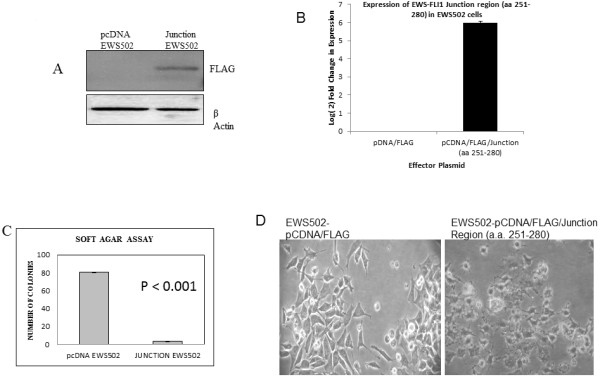
**Over-expression of EWS-FLI1 junction region (aa 251- aa 280) inhibits tumorigenicity, EWS-FLI1 target gene expression and EMT marker genes in Ewing’s sarcoma cells.A**: Confirmation of junction region (aa 251–280) protein expression in transfected EWS502 cell line by immunoblotting with anti Flag antibody. Lane 1: pCDNA/FLAG expressing EWS502 cells, Lane 2: pCDNA/FLAG/Junction (aa 251–280) expressing EWS502 cells. Figure 5**B**: Real time RT-PCR analysis of Junction region (aa 251–280) in pCDNA/FLAG and pCDNA/FLAG/Junction (aa 251–280) stably expressing EWS502 cells. The real time RT-PCR analysis was performed using the same primer set used to clone the junction region and SYBR green chemistry. Figure 5**C**: Soft agar assay showing significant reduction in colony number in EWS502 transfected with junction deletion construct, compared to vector control and wild type EWS502. Figure 5**D**: Photographs of live pCDNA/FLAG and pCDNA/FLAG/Junction (aa 251–280) stably expressing EWS502 cells in culture obtained at 10X magnification.

In order to explore the biological effects of junction region a smaller portion comprising of thirty amino acids (251 to 280) was ectopically expressed in EWS502 Ewing’s Sarcoma cell line. The stable expression of the junction region was confirmed by immunoblotting for the presence of FLAG tagged junction region. (Figure 5A). The over expression was further confirmed by real-time PCR analysis which indicated an Log(2) fold increase of 5.96 in over expressing cells (Figure 5B). When soft agar assay was performed on these cells we found a marked reduction in the colony forming propensity of EWS502 cells in the presence of junction construct, p-value <0.001 (Figure 5C), this indicated that the over expression of junction construct could inhibit anchorage independence and hence tumorigenicity of Ewing sarcoma cells.

Junction construct transfected EWS502 cells underwent marked morphologic changes *in vitro*, adopting a round, rather than the elongated phenotype of empty vector transfected counterpart (Figure 5D).

**Figure 6:**
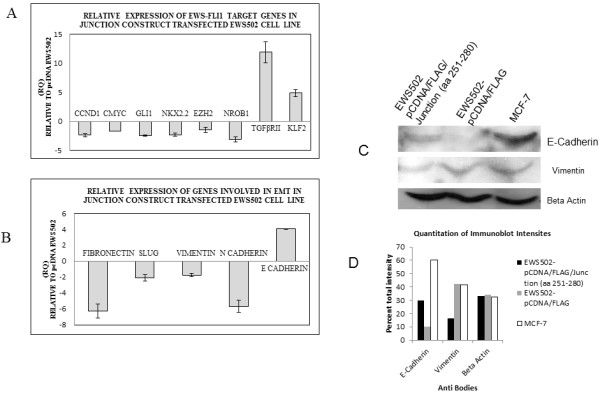
**Real Time PCR for EWS-FLI1 target genes and genes involved in EMT:A: Real time PCR for target genes showing down regulation of EWS-FLI1 regulated genes like Cyclin D1, c-MYC, GLI1, NKX2.2, EZH2, NROB1 in junction construct (aa 251–280) transfected EWS502 cell line relative to vector control pcDNA-EWS502.** Figure 6B: Real Time PCR for EMT markers showing down regulation Fibronectin, Slug, Vimentin, N-Cadherin and up regulation of E-Cadherin in junction region transfected EWS502 cell line relative to vector control pCDNA-EWS502. Figure 6C: Immunoblot analysis of E-Cadherin, Vimentin and Beta Actin expression in MCF-7, pCDNA/FLAG and pCDNA/FLAG/Junction (aa 251–280) stably expressing EWS502 cells. Figure 6D: Quantitation of Immunoblot intensities. The individual intensities are presented as percent total intensity . The total intensity was obtained by adding all the intensities from the individual bands.

Marked decrease in the colony forming ability observed led us to study the expression of the known EWS-FLI1 regulated targets. Real time quantitative RT-PCR analysis of EWS-FLI1 up-regulated target genes were found to be down-regulated in junction construct (a.a. 251–280) transfected cell line - GLI1 by 2.47 fold, NKX2.2 by 2.3 fold, NROB1 by 3.15 fold, Cyclin D1 by 2.4 fold, c-MYC 1.69 by fold, and EZH2 by 1.48 fold compared to pcDNA-EWS502 vector control cell line (Figure 6A). Known EWS-FLI1 down-regulated targets like TGFβRII, and KLF2 showed up-regulation of 11.9 fold and 4.85 fold respectively in junction construct transfected EWS502 cells, compared to vector control.

### Epithelial to mesenchymal transition (EMT) marker genes expression is repressed in the junction construct (aa 251 to aa 280) over-expressing cells

The suppression of tumorigenic potential by the junction construct (aa 251–280) transfected cells also led us to study the effect on expression of EMT markers.

Mesenchymal markers were found to be down regulated (Figure 6B) in junction construct transfected EWS502 cell line. Fibronectin (6.26 fold), Vimentin (1.75 fold) and N Cadherin (5.69 fold) were down regulated compared to vector control*.* EMT promoting gene Slug was also found to be down regulated by 2.1 fold (Figure 6B).

Epithelial marker E-Cadherin showed 4 fold up regulation in junction construct transfected EWS502 (compared to vector control). Immunoblotting was performed to assess the protein expression levels of E-Cadherin and Vimentin relative to pCDNA/FLAG expressing cells. Cell lysate from MCF-7 cells were used as positive control for the expression of E-Cadherin and Vimentin and Beta Actin expression as a control for protein loading. The analysis showed a distinct increased expression of E Cadherin and repression of Vimentin expression in junction construct (aa 251–280) expressing cells (Figure 6C and D). The expression levels assessed for EMT marker genes further indicate the potential of the junction construct (aa 251–280) to repress tumorigenic properties Ewing’s sarcoma cells.

## Discussion

EWS-FLI1 is an ideal therapeutic target protein in Ewing’s sarcoma due to its causative role in the process of tumorigenesis [15,16]. Structural and functional characterization of EWS-FLI1 is therefore important to specifically target this protein which is very likely to benefit patients with Ewing’s sarcoma. Deeper understanding of its sequence structure relationships promises to enable design of novel therapeutic molecules.

EWS-FLI1 is an intrinsically disordered protein and structural property of this protein is less characterized due to its insolubility. In the present study, we modelled the EWS-FLI1 of type 1 fusion type in-silico and identified potential ligand binding sites. The insolubility of the full length EWS-FLI1 could not be overcome despite the widely used solubility tags like Trx and GST. Recovery of the protein in denaturing conditions and refolding was not attempted in this study because studies done previously show, purified EWS-FLI1 and refolded EWS-FLI1 exhibiting differences in orientation of aromatic side chains on exposure to solvent [17]. We therefore identified the ordered region within the EWS-FLI1 structure which had the junction region specific to EWS-FLI1 type 1 and characterized it by circular dichroism (CD).

The junction region of any oncogenic fusion protein is unique for targeting and we, for the first time found the junction region (aa 251–343) of EWS-FLI1 fusion protein to be soluble. Protein solubility under physiological conditions is a prerequisite for drug development, though previous reports [17] have shown the insolubility of the EWS-FLI1, our findings show that the junction region can be obtained in the soluble form and can be used for further structural exploration, with a potential to be a drug target.

Further work needs to be carried out to understand the transition from a disordered to an ordered state by performing CD experiments with titrations against possible interacting proteins or other molecules (for e.g. Promoter Oligonucleotides) regulated by this fusion protein [18-20]. This could be useful in obtaining a crystal structure for the junction region of this fusion protein which would enable identifying a unique target.

As per our knowledge we are the first to describe the dominant negative behaviour of the junction construct (aa 251 – 280). Previous study [7] with EWS-FLI1 deletion mutants showed that both EWS and FLI1 domains are necessary for transformation and deletion of either the EWS domain or the FLI1 DNA-binding domain totally abrogates the transforming ability. In our study we found that the junction region behaved in a dominant negative manner by suppressing tumorigenicity of EWS502 Ewing’s Sarcoma cell line in the soft agar assay. This attenuation could be an indirect effect due to competition for the molecular targets between the wild type EWSFLI1 present in EWS502 and junction region protein which was ectopically overexpressed, or junction region protein interacting with the full length wild type EWS-FLI1 rendering inactive complexes between the two forms of protein.

Because previous studies [21], have suggested that transcriptional activation is critical to the function of EWS-FLI1 as an oncoprotein, we focused our efforts on genes that were up regulated by the fusion protein. Quantitative Real Time PCR was done to check the expression level of some of the well known EWS-FLI1 up regulated target genes in junction construct transfected EWS502 cell line. EWS-FLI1 knockdown based studies and transcriptional profiling data [22] of various Ewing’s sarcoma cell lines have shown that gene like NR0B1, NKX2.2, EZH2, GLI1 are up regulated by EWS-FLI1. Functional studies revealed that ongoing expression of these genes are required for the transformed phenotype of Ewing’s sarcoma and reduction of any of these genes either in transcript level or in protein levels resulted in a significant reduction of oncogenic transformation in various Ewing’s sarcoma cells [23-26]. This is in agreement with our results. In the present study we found more than 2 folds down regulation of NR0B1, NKX2.2 and GLI1 in EWS502 cell line in the presence of junction construct (compared to vector control) which could be the probable reason for the reduced tumorigenicity of junction construct transfected EWS502 cell line *in vitro.*

Previous studies have shown that TGFβRII is a major down regulated target of EWS-FLI1 oncoprotein [27] and introduction of normal TGFβRII into Ewing’s sarcoma cell lines restores TGFβ sensitivity and blocks tumorigenicity. We observed a significant up regulation in TGFβRII and KLF2 in the junction construct transfected EWS502 cells, which again supports the reduced tumorigenicity of junction construct transfected EWS502 cell line.

Earlier studies have shown that Ewing’s sarcoma-specific EWS-ETS oncoproteins were capable of activating Cyclin D1 promoter in transient transfections of tissue culture cells [28,29]. Strong oncogenes such as c-MYC and Cyclin D1 appear to be up regulated by EWS-FLI1 in some models [30,31]. In our study we found 2.4 folds down regulation of Cyclin D1 and 1.48 fold down regulation of c-MYC in junction construct transfected EWS502 compared to vector control.

Imunohistochemistry based studies on EMT markers in earlier studies have shown that Ewing’s sarcoma cells are positive for Vimentin [32]. Ewing's sarcoma cell lines produce a complex extra cellular matrix containing Fibronectin [33]. Previous studies have shown that Slug expression triggers EMT and its expression is inversely correlated with E-Cadherin expression [34]. In the present work we have suggested that down regulation of mesenchymal markers and up regulation of epithelial marker like E-Cadherin in EWS502 cell line, in the presence of junction construct may be a probable reason for the suppression of tumorigenicity *in vitro.*

## Conclusions

We for the first time have identified a region encompassing the junction region of EWS-FLI1 protein to be part of putative small molecule binding sites (aa 251 – 343) and this region can be expressed in the soluble form which could pave the way for structural characterization of this region. Our study also indicates a propensity for a short junction construct (aa 251 – 280 a 30 amino acid-peptide) to exhibit a dominant negative effect on the functions of wild type EWS-FLI1 protein by inhibiting target gene expression and tumorigenicity *in vitro*.

## Competing interests

Authors declare no conflict of interest.

## Authors’ contributions

BJ performed the cloning and molecular genetic studies and drafting of the manuscript, RV performed sequence and bio-informatics analysis, GG performed the Western blotting and revision of the manuscript, KS performed the in-silico structural analysis, and TR conceptualized the study, analyzed the data and drafted and revised the manuscript. All authors read and approved the final manuscript.

## Pre-publication history

The pre-publication history for this paper can be accessed here:

http://www.biomedcentral.com/1471-2407/12/513/prepub
